# A lower cut-off for lymph node harvest predicts for poorer overall survival after rectal surgery post neoadjuvant chemoradiotherapy

**DOI:** 10.1186/s12957-020-01833-8

**Published:** 2020-03-20

**Authors:** Charleen Shanwen Yeo, Nicholas Syn, Huimin Liu, Sau Shung Fong

**Affiliations:** 1 Department of General Surgery, Tan Tock Seng Hospital, 11 Jalan Tan Tock Seng, Singapore, 308433 Singapore; 2 Yong Loo Lin School of Medicine, National University of Singapore, Singapore, Singapore; 3 Raffles Surgery Centre, Raffles Hospital, Singapore, Singapore

**Keywords:** Rectal cancer, Neoadjuvant, Chemotherapy, Radiotherapy, Lymph node

## Abstract

**Background:**

A lymph node harvest (LNH) of < 12 is a predictor for poor prognosis in rectal cancer patients. However, neoadjuvant chemoradiotherapy (NACRT) is known to decrease LNH; hence, a cut-off of 12 is inappropriate in such patients. This paper aims to establish a LNH cut-off predictive for disease-free and overall survival in NACRT patients.

**Methods:**

A retrospective review of patients who underwent elective surgery for rectal cancer from 2006 to 2013 was performed. All patients with R1/2 resections and presence of metastases and those operated on for recurrence were excluded. Patient demographics, clinical features, operative details, LNH, 30-day mortality and disease-free and overall survival were recorded. *P* values of < 0.05 were considered significant.

**Results:**

A total of 257 patients were studied, with 174 (68%) males and a median age of 66 years. Ninety-four (37%) patients received long-course NACRT, and 122 (48%) patients were stage 2 and below. Median LNH was 17, which was reduced in the NACRT group (14 versus 23, *P* < 0.01). Average length of stay was 9 ± 8 days, with a major post-operative complication rate of 4%. Using hazard ratio plots for the NACRT subgroup, LNH cut-offs of 16.5 and 8.5 were obtained for disease-free survival (DFS) and overall survival (OS) respectively. Survival analysis showed that a LNH cut-off of 8.5 was a significant predictor of OS (*P* < 0.001).

**Conclusion:**

LNH is reduced in patients receiving NACRT before rectal cancer surgery. A LNH of 9 and above is associated with improved overall survival. We propose that this can be used as a tool for prognosis.

## Introduction

The number of harvested lymph nodes (LN) is a well-established prognostic factor in patients with rectal cancer. The American Joint Committee on Cancer (AJCC) recommends that at least 12 LN are needed to confirm node-negative disease for rectal cancer [1, 2].

Neoadjuvant chemoradiotherapy (NACRT) is known to decrease the lymph node harvest (LNH) in the resected specimen [3–5]. While studies have shown that LNH is associated with recurrence and survival in rectal cancer patients who have undergone upfront surgery [6, 7], its prognostic value in patients who have received NACRT is debatable [8–13]. Although some studies show that LNH has no significant correlation with recurrence or survival in NACRT patients [9, 12], some report that a suboptimal LNH of < 12 independently predicts worse overall survival irrespective of neoadjuvant treatment [11, 13]. A low LN count could represent either poor sampling of the draining LN basin and hence inadequate staging or a good response to neoadjuvant treatment and therefore a surrogate marker for improved survival. Currently, the National Comprehensive Cancer Network (NCCN) guidelines indicate that a minimal LNH of 12 is no longer applicable for patients who have undergone neoadjuvant therapy.

Hence, the aim of this project is to determine a new LNH cut-off in the NACRT subgroup for both disease-free and overall survival in our patient population.

## Methodology

This was a single-centre retrospective review of all patients who underwent elective surgery for rectal cancer in our institution from January 2006 to December 2013. Inclusion criteria were all patients who underwent elective surgical resection for histologically proven rectal adenocarcinoma. Exclusion criteria are as follows: all patients with R1/2 resections, involvement of the circumferential radial margin, high rectal tumours (defined as tumours for which a high anterior resection and partial mesorectal excision was performed), presence of metastases, emergency surgeries, and those operated on for recurrence. This study was conducted with the approval of our institution’s ethical review board (Domain Specific Review Board reference number: 2018/00540). We chose a period of study before 2014 to allow for sufficient follow-up for survival analysis.

### Pre-operative conduct

All patients had a pre-operative diagnosis of rectal cancer based on endoscopically obtained biopsy with histological confirmation of the primary tumour. All patients received a staging computed tomography (CT) scan of the thorax, abdomen and pelvis, as well as a magnetic resonance imaging (MRI) of the rectum prior to surgery. Patient demographic details and tumour characteristics were recorded accordingly. Patients in the NACRT group received long-course neoadjuvant chemoradiotherapy—comprising 45 Gy of radiotherapy given in 25 fractions over 5 weeks, with concomitant oral capecitabine 825 mg/m^2^ twice daily as a radiosensitiser. All patients were discussed at the multidisciplinary tumour board. Patients with T3-, T4- or N-positive disease on pre-operative MRI were selected to undergo NACRT. In this group of patients, upfront surgery may still proceed if the surgeon determines that the circumferential resection margin was sufficiently wide to reduce the risk of a R0 resection or if the patient declines neoadjuvant treatment.

### Operative details

All surgeries were performed by four specialists in the colorectal subspecialty service in our institution. Surgeries were performed either using the traditional open technique or via the laparoscopic approach, according to the discretion of the primary consultant and in consultation with the patient. If NACRT was given, surgery was performed within 6–10 weeks from completion of chemoradiotherapy. Oncologic en bloc resection of the specimen was performed, along with total mesorectal excision to ensure adequate clearance of the draining lymphovascular basin. All resected specimens were sent for histopathological assessment. Lymph node retrieval and assessment were performed by trained pathologists from our institution. In order to maximise the lymph node yield, all pathologists performed chemical fat clearance and took additional random sections of mesorectal fat for sampling. In cases with less than 12 lymph nodes found during the initial assessment, a repeat examination was performed by a second pathologist.

### Outcome measures

All patients were followed up for at least 5 years post-operatively. Length of stay, 30-day mortality, post-operative complications, disease-free survival (DFS) and overall survival (OS) were calculated for all patients. All patients who received neoadjuvant therapy also received adjuvant chemotherapy. Patients were given 3-month follow-up clinic visits for the first 2 years, including physical examination and CEA levels. Patients were then seen 6 months from the third year onwards, until the 5th post-operative year. Surveillance colonoscopies were performed at 1st, 3rd and 5th year post-surgery, then 3–5 years thereafter. For patients with an incomplete scope pre-surgery, completion colonoscopy was performed within 6 months post-surgery to rule out synchronous lesions. Surveillance CT scans (thorax, abdomen and pelvis) were performed at 12-month intervals until the 5th post-operative year.

Disease-free survival was defined as the time from surgery to death or disease progression, and patients without these events were censored at last follow-up. Overall survival was defined as the time from surgery to death from any cause, and patients who were alive at last follow-up were censored.

LNH was categorised into < 12 and ≥ 12 based on AJCC and UICC guidelines of 12 being the optimal LNH cut-off in resected specimens.

### Statistical analysis

Chi-square test, Mann-Whitney *U* test, Kruskal-Wallis test and multiple linear regression analyses were used as appropriate. For the NACRT subgroup, we investigated the relationship between LNH and survival outcomes by iteratively dichotomizing the LNH at each integer value and computing hazard ratios at each cut-off value. The optimal LNH cut-off was determined based on the cut-off which was associated with the most significant split in Kaplan-Meier curves (i.e. smallest univariable log-rank *P* value). Kaplan-Meier survival curves were then plotted based on these LNH cut-offs for both DFS and OS respectively. *P* values of < 0.05 were considered significant. All statistical analysis was performed using SPSS version 21.0 and R software version 3.4.2 (The R Foundation for Statistical Computing).

## Results

### Clinical characteristics and operative details

There were a total of 257 patients included in this study, with 174 (68%) males and a mean age of 66 ± 11 years old. The rectal tumours were at a mean distance of 6 ± 3 cm from the anal verge.

Ninety-four (37%) patients received pre-operative NACRT. With regard to the type of surgery, there were 32 (12%) ultra-low anterior resections, 168 (65%) low anterior resections, 19 (7.4%) Hartmann’s procedure and 38 (15%) abdominoperineal resections. One hundred fifty (58%) patients underwent laparoscopic surgery, while 107 (42%) patients underwent open surgery. One hundred twenty-two (48%) of cancer patients were stage 1 or 2, while the remaining 135 (52%) were stage 3.

When stratified according to the upfront surgery versus NACRT, a significantly higher proportion of patients in the NACRT group had lower rectal tumours (mean distance of 5.5 versus 7.0 cm from anal verge, *P* < 0.001). Accordingly, a greater proportion of patients in the NACRT group also underwent abdominoperineal resections or ultra-low anterior resections as compared to the upfront surgery group (Table 1).

**Table 1:** Clinicopathologic characteristics and outcomes stratified by neoadjuvant treatment (values in either median ± SD or no. (%) unless otherwise stated)

Variable	Category	Total (*N* = 257)	Upfront surgery (*N* = 163)	NACRT group (*N* = 94)	*P* value
Age at diagnosis (years)		66 ± 11	67 ± 11	64 ± 10	0.132
Gender	Male	174 (68)	106 (65)	68 (72)	0.227
	Female	83 (32)	57 (35)	26 (28)	
Distance from anal verge (centimetres)		6 ± 3	7.0 ± 3.4	5.5 ± 2.4	**< 0.001**
Type of operation	Ultra-low anterior resection	32 (12)	16 (10)	16 (13)	**0.001**
	Low anterior resection	168 (65)	117 (72)	51 (65)	
	Hartmann’s procedure	19 (8)	15 (9)	4 (7)	
	Abdominoperineal resection	38 (15)	15 (9)	23 (15)	
Lymph node harvest		17 ± 11	23 ± 13	14 ± 6.7	**< 0.001**
Lymph node harvest	< 12	53 (21)	17 (10)	36 (38)	**0.002**
	≥ 12	204 (79)	146 (90)	58 (62)	
Post-operative complications	None	225 (88)	140 (86)	85 (88)	0.729
	Clavien-Dindo 1 and 2	27 (11)	19 (11)	8 (10)	
	Clavien-Dindo 3 and above	5 (1)	4 (3)	1 (2)	
Length of stay (days)		9 ± 8	10 ± 8	8 ± 8	0.190
30-day mortality	Yes	255 (99)	2 (1)	0 (0)	0.281
	No	2 (1)	161 (99)	94 (100)	
Recurrence	Yes	60 (23)	40 (25)	20 (21)	0.551
	No	197 (77)	123 (75)	74 (79)	
Disease-free survival (months)		66 ± 41	62 ± 40	73 ± 42	0.067
Mortality	Yes	90 (35)	60 (37)	30 (32)	0.428
	No	167 (65)	103 (63)	64 (68)	
Overall survival (months)		71 ± 38	68 ± 38	77 ± 39	0.059

*NACRT* neoadjuvant chemoradiotherapy

### Operative outcomes

There were 5 (2%) patients with post-operative complications of Clavien-Dindo classification 3 and above, of which there were 4 anastomotic leaks and 1 intestinal obstruction. The median length of stay was 9 ± 8 days, and 30-day mortality incidence was 0.8% (*n* = 2). Twenty-three percent (*n* = 60) of patients had recurrence within 5 years post-surgery, of which 11 (18%) were locoregional, 41 (68%) were systemic and 8 (13%) were with both locoregional and systemic. The overall 5-year disease-free survival was 66 ± 41 months. For the 5-year duration of follow-up analysed, the overall mortality was *n* = 90 (35%), with an overall survival of 71 ± 38 months.

There was no statistically significant difference between the upfront surgery and NACRT group in terms of operative outcomes (Table 1). There was also no statistically significant difference between the lymph node yield when stratified according to the laparoscopic versus open surgery (17 versus 16, *P* = 0.56).

### Impact of NACRT on LNH

An overall median of 17 ± 11 lymph nodes were retrieved. Patients who received NACRT had a significantly reduced LNH compared to those that had upfront surgery (14 ± 7 versus 23 ± 13, *P* < 0.001). Similarly, 90% (*n* = 146) of patients with upfront surgery met the recommended LNH cut-off of ≥ 12, versus only 62% (*n* = 58) in the NACRT group.

### Factors associated with DFS and OS

Table 2 shows the multivariate analysis of clinicopathological factors on disease-free and overall survival. On multivariate analysis, tumour staging was the only significant prognostic variable for both DFS and OS, with lower stage 1 and 2 tumours having a longer DFS and OS versus stage 3 tumours. Patients in the LNH < 12 subgroup had a decreased DFS and OS compared to the LNH ≥ 12 subgroup, although this was not statistically significant (DFS = 61 versus 62 months, OS = 68 versus 73 months). Table 3 shows the subgroup analysis with separation of patients into the NACRT versus upfront surgery, and looks into factors related to tumour staging and lymph node yield alone. When stratified according to the NACRT versus upfront surgery, tumour staging remained significantly associated with a longer disease-free and overall survival. However, during subgroup analysis of patients who received upfront surgery, our data shows that a LNH of 12 and above was significantly associated with a longer disease-free and overall survival, with *P* values of 0.040 and 0.013 respectively (Table 3). Contrastingly, LNH > 12 was not a significant predictor of long-term outcome for the NACRT subgroup.

**Table 2:** Multivariate analysis of clinicopathologic factors related to disease-free and overall survival

Variables	Category	*N*	Disease-free survival		Overall survival	
			Median ± SD (months)	*P* value	Median ± SD (months)	*P* value
Age at diagnosis (years)	< 50	18	81 ± 46	0.180	90 ± 38	0.370
	50–64	101	68 ± 42		73 ± 39	
	65–74	74	63 ± 39		69 ± 37	
	> 75	64	63 ± 39		65 ± 38	
Gender	Male	174	63 ± 42	0.160	69 ± 39	0.470
	Female	83	73 ± 38		76 ± 36	
Neoadjuvant therapy	No	163	62 ± 40	0.060	68 ± 38	0.191
	Yes	94	73 ± 41		77 ± 42	
Type of operation	Ultra-low anterior resection	32	56 ± 38	0.908	61 ± 39	0.659
	Low anterior resection	168	67 ± 43		73 ± 40	
	Hartmann’s procedure	19	55 ± 42		59 ± 38	
	Abdominoperineal resection	38	77 ± 44		59 ± 42	
Tumour staging	Stage 1	61	84 ± 39	**< 0.001**	85 ± 40	**< 0.001**
	Stage 2	61	67 ± 35		72 ± 38	
	Stage 3	135	58 ± 38		64 ± 37	
Lymph node harvest	< 12	53	61 ± 44	0.070	62 ± 42	0.051
	≥ 12	204	68 ± 42		73 ± 36

**Table 3:** Subgroup analysis of neoadjuvant chemoradiotherapy (NACRT) and upfront surgery patients

Variables	Category	*N*	Disease-free survival		Overall survival	
			Median ± SD (months)	*P* value	Median ± SD (months)	*P* value
**NACRT group (*N*= 94)**						
Tumour staging	Stage 1	18	88 ± 39	**0.026**	88 ± 39	**0.051**
	Stage 2	32	73 ± 40		76 ± 39	
	Stage 3	44	67 ± 37		74 ± 36	
Lymph node harvest	< 12	36	61 ± 38	0.801	63 ± 41	0.436
	≥ 12	58	80 ± 40		86 ± 39	
**Upfront surgery group (*N*= 163)**						
Tumour staging	Stage 1	43	82 ± 38	**0.001**	84 ± 41	**0.003**
	Stage 2	29	61 ± 37		68 ± 40	
	Stage 3	91	54 ± 35		60 ± 37	
Lymph node harvest	< 12	17	61 ± 38	**0.040**	61 ± 39	**0.013**
	≥ 12	146	63 ± 41		68 ± 38	

### Optimal LNH cut-offs for the NACRT group

**Fig. 1:**
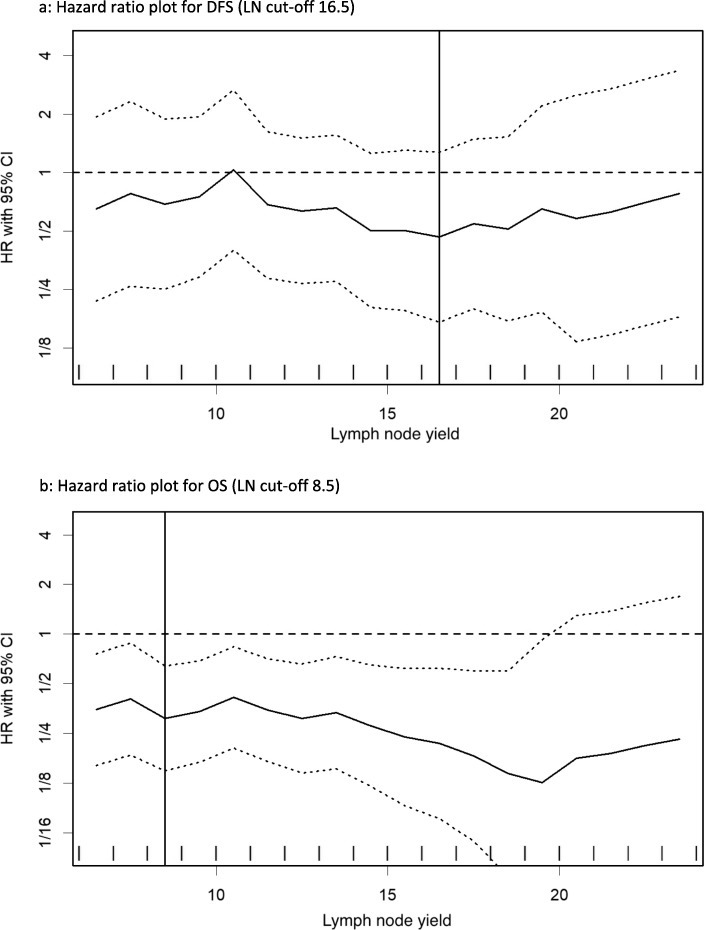
Hazard ratio plots to determine optimal lymph node (LN) cut-offs for disease-free survival (DFS) and overall survival (OS) in the neoadjuvant chemoradiotherapy (NACRT) group. **a** Hazard ratio plot for DFS (LN cut-off 16.5). **b** Hazard ratio plot for OS (LN cut-off 8.5)

**Fig. 2:**
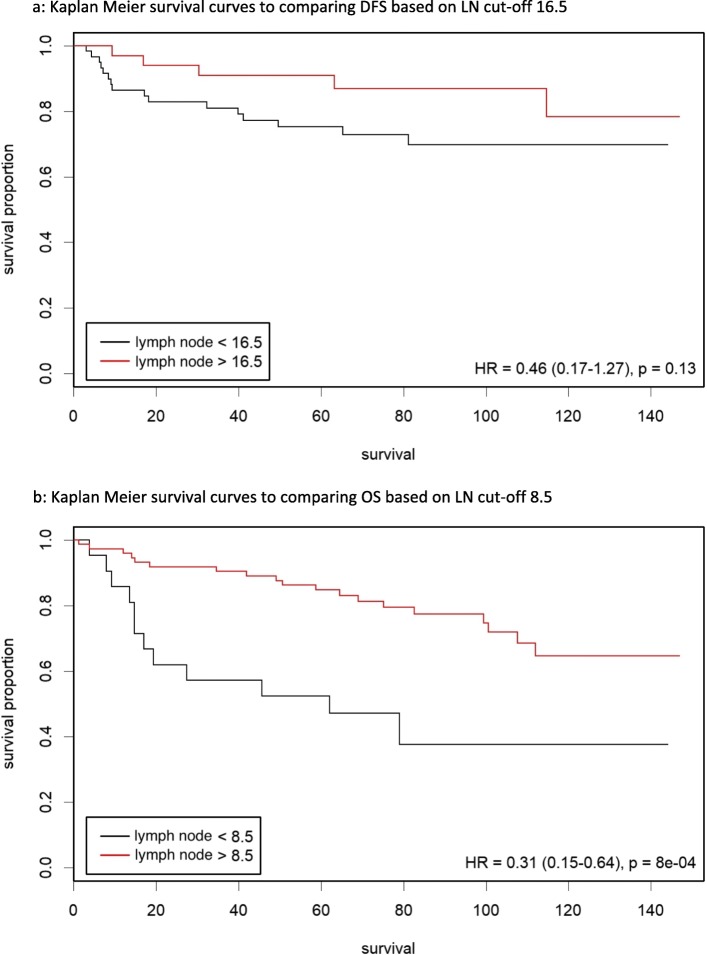
Kaplan-Meier survival curves to compare disease-free survival (DFS) and overall survival (OS) based on lymph node (LN) cut-offs in the neoadjuvant chemoradiotherapy (NACRT) group. **a** Kaplan-Meier survival curves to comparing DFS based on LN cut-off 16.5. **b** Kaplan-Meier survival curves to comparing OS based on LN cut-off 8.5

As our data shows that NACRT significantly reduces LNH and that a cut-off of 12 was not applicable for this subgroup, we proceeded on to establish if there was a new optimal LNH cut-off for both DFS and OS in NACRT patients. Hazard ratio plots demonstrate that the optimal LNH cut-off for DFS and OS are 16.5 and 8.5 respectively (Fig. 1). The Kaplan-Meier curve survival analyses show that a LNH cut-off of 8.5 was a significant predictor for OS (HR 0.31, 95% CI 0.15–0.64, *P* < 0.001); however, a LNH cut-off of 16.5 did not significantly predict DFS (HR 0.46, 95% CI 0.17–1.27, *P* = 0.13) (Fig. 2).

Using this new LNH cut-off of 9, we proceeded on to further subgroup analysis of the NACRT cohort. Table 4 demonstrates that on the multivariable analysis, a LNH cut-off of 9 was a significant predictor for both disease-free and overall survival.

**Table 4:** Subgroup multivariate analysis of clinicopathologic factors related to disease-free and overall survival for the neoadjuvant chemoradiotherapy (NACRT) group, with a new LN cut-off of 9

Variables	Category	*N* = 94	Disease-free survival		Overall survival	
			Median ± SD (months)	*P* value	Median ± SD (months)	*P* value
Age at diagnosis (years)	< 50	7	82 ± 38	0.236	91 ± 35	0.082
	50–64	44	65 ± 41		72 ± 40	
	65–74	26	63 ± 40		67 ± 36	
	> 75	17	62 ± 33		64 ± 38	
Gender	Male	68	64 ± 35	0.770	68 ± 40	0.835
	Female	26	72 ± 36		74 ± 38	
Type of operation	Ultra-low anterior resection	51	56 ± 35	0.838	60 ± 41	0.907
	Low anterior resection	16	67 ± 40		72 ± 40	
	Hartmann’s procedure	4	57 ± 41		61 ± 37	
	Abdominoperineal resection	23	74 ± 39		60 ± 36	
Tumour staging	Stage 1	18	82 ± 40	**0.008**	86 ± 35	**0.012**
	Stage 2	32	64 ± 37		60 ± 37	
	Stage 3	44	53 ± 37		57 ± 38	
Lymph node harvest	< 9	21	52 ± 36	**0.028**	55 ± 39	**0.009**
	≥ 9	73	63 ± 45		72 ± 40	

## Discussion

Our study shows that NACRT results in a significant reduction in LNH as compared to upfront surgery. This finding is well supported by the current literature [3–5], with prior large-scale retrospective studies by Ha et al. [4] and Amajoyi et al. [5] demonstrating mean LNH of 14.5 and 9 respectively in the neoadjuvant group, versus 21.5 and 13 in the upfront surgery group. A meta-analysis by Mechera et al. also shows that NACRT decreases the LNH by approximately two to four lymph nodes [14]. It has been suggested that this is due to a reduction in lymph node size due to apoptosis and involution induced by chemoradiotherapy [4, 15, 16]. Given that NACRT significantly reduces LNH, the utility of LNH as a prognostic factor in such patients should be interpreted with caution.

Traditionally, a cut-off of 12 lymph nodes is used as an indicator of adequate oncological clearance, in order to confirm node-negative rectal cancer as per AJCC guidelines [1, 2]. However, LN yield is known to be affected by pre-operative NACRT [3–5], and it is proposed that the traditional cut-off of 12 should not be applied to this subgroup of patients. The current NCCN guidelines do not give a guideline of a minimum number of harvested nodes in patients who have undergone neoadjuvant therapy. A low LNH in NACRT patients could either represent either poor oncological clearance and hence inadequate staging or a good response to NACRT and therefore a predictor of good outcome. While some studies show that LNH predicts worse overall survival regardless of NACRT [11, 13], majority of the literature are proponents of the notion that a LNH metric may not be clinically relevant in the era of neoadjuvant therapy [9, 12, 17, 18]. Our paper demonstrates that a LNH cut-off of 12 is not significantly associated with poorer DFS and OS in patients who received pre-operative NACRT. However, further survival curve analysis within the NACRT group demonstrates that a cut-off of 8.5 significantly predicted overall survival.

As NACRT significantly reduces LNH in rectal cancer patients undergoing surgical resection, it is important for surgeons to recognise that when using LNH as a prognosticating factor. These two groups of patients—upfront surgery versus NACRT—should be evaluated using a different metric. Establishing a new LNH cut-off in NACRT patients is important as it allows clinicians to establish a more accurate surrogate judgement for the quality of their surgery, as well as better counsel their patients regarding future prognosis. In some patients who have concerns about undergoing advised completion chemotherapy after NACRT, having a lymph node yield of < 9 provides added reason to proceed. Conversely, in elderly patients who have a lymph node yield of less than 12 but more than 9 after NACRT, consideration should also be taken as to whether completion chemotherapy will be beneficial. Given the increasing prevalence of colorectal cancer in older adults [19], studies have shown that the elderly are less likely to be recommended adjuvant therapy in view of significant side effects and limited survival benefit [20, 21]. Lastly, our results are especially pertinent to our local population, as it allows us to apply these results to patients from a similar background. A recent local paper published by Chan et al. demonstrated that the LNH cut-off of 12 had no significant impact on overall survival in patients who received neoadjuvant therapy [22]. Perhaps, a new lower cut-off of 9 can be used in our local population instead.

As per all retrospective reviews, the authors acknowledge that there are limitations to this paper. The variables analysed can only be associated with the outcomes, and a directional causality cannot be proven. The treatment modality was also patient and surgeon dependent; hence, the allocation of patients into the two treatment groups was not entirely random. However, this study has a long follow-up interval of 5 years, with a sizeable patient population. More prospective longer-term studies are needed to establish if a new LNH cut-off of 9 and above should be used as a surrogate marker for better patient outcome instead.

## Conclusion

Instead of the traditionally accepted cut-off value of 12 lymph nodes, perhaps a lower number can be considered optimal in NACRT patients. Our study shows that a LN cut-off of 9 and above can be used to predict improved overall survival in NACRT patients. This data will help clinicians better prognosticate their patients’ outcomes and may influence their decisions for post-operative management should lymph node yield be insufficient.

## Data Availability

The datasets used and/or analysed during the current study are available from the corresponding author on reasonable request.
